# Assessing the relevance of allergenic pollen in indoor environments—current knowledge base and research needs

**DOI:** 10.1007/s40629-023-00251-y

**Published:** 2023-06-13

**Authors:** Sascha Nehr, Regina M. B. O. Duarte, Antoine S. Almeida, Lukas Baus, Karl-Christian Bergmann

**Affiliations:** 1 CBS International Business School, Kaiserstraße 6, 50321 Brühl, Germany; 2 CESAM—Center for Environmental and Marine Studies, Department of Chemistry, University of Aveiro, 3810–193 Aveiro, Portugal; 3 Charité – Universitätsmedizin Berlin, Freie Universität Berlin, Humboldt-Universität zu Berlin, Charitéplatz 1, 10117 Berlin, Germany

**Keywords:** Indoor pollen, Pollen taxa, Indoor air quality, Health effects, Perennial allergy, Statistical assessment

## Abstract

Airborne pollen allergens—a relevant component of bioaerosols and, therefore, of airborne particulate matter—are considered an important metric in air quality assessments. Although the measurement of airborne pollen allergen concentrations in outdoor environments (namely, in urban areas) has been recognized as a key environmental health indicator, no such obligation exists for indoor environments (dwellings or occupational settings). However, people spend most of their daily time (80–90%) indoors, where the majority of their exposure to air pollution, including pollen allergens, occurs. Nonetheless, the relative importance of airborne pollen allergen exposure indoors differs from outdoors because of differences in pollen loads, sources, dispersion, and degree of penetration from the outdoor surroundings, as well as the differences in the allergenic pollen profiles. In this brief review, we mined the literature over the last 10 years to summarize what existing measurements reveal about the relevance of airborne allergenic pollen in indoor environments. The research priorities on this topic are presented, highlighting the challenges and the motivations for obtaining pollen data in built environments which are key to understand the extent and mechanisms of human exposure to airborne pollen allergens. Thus, we provide a comprehensive assessment of the relevance of airborne allergenic pollen in indoor environments, highlighting knowledge gaps and research needs related to their health effects.

## Need for indoor pollen monitoring and information

As airborne particles of biological origin, pollen is considered the main cause of allergenic rhinitis. In the European Union, pollen-associated allergic diseases cause annual costs on the order of tens of millions of euros related to absence from work (absenteeism) and reduced productivity (presenteeism). These costs need to be covered by the healthcare system and/or society as a whole [1], which is why publicly available pollen forecasts can contribute to the reduction of the burden on public health systems. Beyond application in the healthcare system, the use of pollen data can also support other areas, e.g., agriculture, climatology, and biodiversity research. Moreover, the World Health Organization (WHO) identified allergen exposure—including the presence of pollen—and the interaction with other environmental and behavioral factors as a future research need [2].

Pollen monitoring and information systems are implemented in some European countries. However, the different pollen monitoring and information systems of, e.g., Finland [3], France [4], the United Kingdom [5], Austria [6], Switzerland [7], and Germany [8], vary in funding, administration, number of measurement sites, data processing, and data presentation [9]. For example, while the Swiss system is publicly funded and administrated, other systems are on a voluntary basis. To date, France is the only European country with a statutorily obligation to measure airborne biological particles [10]. Under this scenario, the EUMETNET AutoPollen programme, an initiative of the European National Meteorological and Hydrological Services, was recently established as a platform to develop a European automatic pollen monitoring network [11]. According to Clot et al. [11], the main purpose of AutoPollen is not only to establish long-term automatic pollen monitoring across the different European countries, but also to provide pollen observations and model forecasts to the public, medical practitioners, and health authorities. While systematic pollen observations and forecasts in outdoor environments have already been available for several decades in terms of implementation and data availability, there is no obligation regarding the monitoring of airborne pollen in indoor environments at the European level. Due to the lack of legally binding acts to tackle indoor pollen loads, there are several technical standards to encounter the burden of disease (symptom load) posed by allergenic pollen. Available technical standards mostly address filtration devices in ventilation installations [12–20]. Thus, there is still a demand to improve our understanding of the burden of airborne allergenic pollen in the built environment and to bridge the gap between research and practical applications addressing appropriate mitigation strategies that optimize indoor air quality. Furthermore, scientific findings need to be disseminated directly to relevant stakeholders such as architects, building engineers, building managers, property developers, and urban planners.

Given the fact that humans spend a large part of their time indoors, this review highlights the relevance of studying and monitoring allergenic pollen in indoor environments. It focusses on scientific articles published between 2012 and 2022 to assess information on (i) the presence and annual profile of allergenic pollen in indoor environments, (ii) the quantification of allergenic pollen, and (iii) the correlation with allergy symptoms. A comprehensive assessment of the relevance of airborne pollen in the built environment comprises both the pollen loads (concentrations) and the occupants’ symptoms. On the one hand, data on the pollen concentration alone cannot inform about the health impact on the allergy sufferer. On the other hand, data on the occupants’ symptoms alone cannot inform about the pollen concentration or the corresponding annual concentration profile [21]. Therefore, the present work aims to systematically compile available knowledge combining pollen loads and symptom loads in the built environment, and to highlight knowledge gaps and research needs.

## Methods

The data presented in this brief review on the relevance of airborne allergenic pollen in indoor environments was retrieved from the databases of the Royal Chemical Society, ScienceDirect, Wiley, and Clarivate using the string of key words: “indoor pollen” or “pollen taxa” or “indoor air quality” or “health effects” or “perennial allergy” or “pollen metabolites” or “statistical assessment” or “pollen count” [22–25]. The time period of the literature review was chosen from 2012–2022 to reflect the current evolution of research in this field during the last decade, to identify research gaps, and to derive recommendations for future research directions. From the search results, original research articles were selected for further analysis. These search criteria resulted in 29 documents.

The literature review is focused on the research question: “What is the relevance of airborne allergenic pollen in indoor environments?” To answer this question, the information extracted from the literature survey was grouped into the following five categories:

- Information on the studied area and the building type (WHERE),
- Information on the investigation period (WHEN),
- Information on the pollen speciation (WHAT),
- Information on the number concentration (HOW MUCH), and
- Information on the observed allergy symptoms of the building occupants (WHY).

## Results and discussion

The results of the literature survey are presented in Table 1 grouped into the categories of WHERE, WHEN, WHAT (subsection “Presence and annual profile of allergenic pollen indoors”), HOW MUCH (subsection “Quantification of allergenic pollen”), and WHY (subsection “Correlation with allergy symptoms”) and addressed in more detail in the corresponding subsections.

**Table 1:** Information extracted from the search results grouped into the following categories: WHERE, WHEN, WHAT, HOW MUCH, and WHY

Studied area, building type	Investigation period	Pollen speciation	Pollen number concentration	Observed allergy symptoms/health effects	Reference
Germany, occupational environments	Autumn	No	No	Self-reported prevalence of allergies for age groups, sex, living area, and occupation	[26]
Literature review on indoor/outdoor pollen concentration ratio	Spring, summer, autumn, winter	n/a	n/a	n/a	[27]
USA, university building	Winter	Yes	Yes	–	[28]
Spain, university building	Winter	No	No	–	[29]
USA, public schools and homes	Spring, summer, autumn, winter	No	No	Self-reported health status with respect to asthma and allergy-related symptoms	[30]
Japan, home environments	Spring, summer, autumn, winter	No	No	Association between the onset of sick-building syndrome and occupant characteristics, social and residential environment, and lifestyle	[31]
Denmark, private homes	Spring, summer, autumn, winter	Yes	No	–	[32]
Italy, research center building	Summer and winter	Yes	Yes	–	[33]
Italy, research center building and university building	Winter	No	Yes	–	[34]
Simulated indoor environment, multiscale computational fluid dynamics	Spring	No	No	Assessment of the increased virus transmission rate through airborne coronavirus particles or airborne infected saliva droplets adhering to pollen grains	[35]
China, 30 m^3^ Teflon film reactor	Dry season	No	Yes	–	[36]
Literature review on the allergenic activity of indoor allergens	n/a	n/a	n/a	Inducement of inflammation by pollen allergens through non-IgE-mediated pathways	[37]
Singapore, home environment (bedroom)	n/a	No	Yes	–	[38]
Spain, public transport (train)	Spring	Yes	Yes	–	[39]
Literature review on the assessment of bioaerosol exposure	n/a	n/a	n/a	n/a	[40]
Germany, environmental research station	Spring to summer	Yes	Yes	Investigation of nasal and pulmonary symptoms in grass pollen allergic volunteers	[41]
Literature review on the current status related to chemical indoor air pollution in healing spaces	n/a	n/a	n/a	n/a	[42]
China, hospital	Summer	No	No	Investigation of symptoms of allergic rhinitis in patients sensitive to *Artemisia* pollen through a double-blind, clinical controlled trial with active and inactive air purifiers	[43]
Poland, n/a	n/a	No	No	Association between sensitization to seasonal allergens (e.g., timothy or birch) as well as perennial allergens (e.g., house dust mites and cats) with asthma and allergenic rhinitis in children	[44]
Austria, office building	Winter	Yes	Yes	–	[45]
Spain, indoor rooftop greenhouse	Winter to spring	Yes	Yes	–	[46]
n/a, mobile exposure chamber	n/a	No	No	Development of nasal, ocular or bronchia irritations through the exposure to grass pollen	[47]
USA, private homes	Spring	Yes	No	–	[48]
Finland, university buildings, nursery schools and farmhouse	Winter and summer	Yes	No	–	[49]
Germany, university building	Spring	Yes	Yes	–	[50]
Austria, private home	Winter to summer	Yes	No	–	[51]
France, private homes	Winter to summer	Yes	Yes	–	[52]
Austria, private home	Spring to autumn	Yes	Yes	–	[53]
India, market, saw mill, poultry shed and cow shed	Spring to winter	Yes	Yes	–	[54]

*n/a* not applicable

### Presence and annual profile of allergenic pollen indoors

As summarized in Table 1, the body of literature addressing indoor airborne allergenic pollen concentration and seasonality has a strong geographic focus in Europe and the United States, with only five studies being conducted in Japan, China, Singapore, and India [31, 36, 38, 43, 54]. The lack of data for southern hemisphere regions clearly suggests that similar studies in this geographic sector are urgently needed to provide a much broader global picture of the presence and annual profile of indoor allergenic pollen. As pollen seasonal magnitude is strongly influenced by regional biogeography, it is very likely that the indoor allergenic pollen profile in southern hemisphere regions and its health effects differ from those of northern hemisphere regions. Furthermore, most of the investigated sites are either educational or research facilities, most likely due to the fact that these indoor locations bring together a high number of people (students, teachers, and assistant personnel), for whom suitable working and learning conditions and their ventilation are of utmost importance. Results from the investigation of private homes [30–32, 38, 48, 51–53], an office building [45], occupational settings (market, saw mill, agriculture) [54], a Teflon film reactor [36], and a mobile exposure chamber [47] are presented in dedicated studies. Nevertheless, one cannot neglect the importance of also focusing on a diversity of pollen conditions and, therefore, on indoor environments that people are subjected to. Thus, the importance of accessing high-quality and detailed information on the presence and profile of allergenic pollen in different indoor settings is key to develop preventive measures to avoid exposure to aeroallergens.

When information is made available, most studies on educational and research facilities, private homes as well as occupational settings are characterized by a good seasonal coverage throughout the course of a year. As the indoor pollen exposure results from outdoor pollination, it is evident that there is a temporal relationship between both phenomena, and that there is a potential risk to allergic people to develop symptoms outside the pollination period due to pollen that has accumulated and settled in indoor environments [52]. A study on pollen loads in public transport even showed the extension of the pollen season of some taxa (i.e., *Platanus, Urticaceae*) for several days in indoor environments (trains). This indicates that closed transport environments can act as biological pollution reservoir which could have consequences on the incidence of passengers’ health [39].

According to Pichot et al., the increment of pollen penetration coefficients from winter to summer strongly supports the hypothesis that the opening of doors and windows facilitates the penetration of pollen into dwellings [52]. Also important is the evidence of the impact of increasing climate change scenarios on aeroallergen production and atmospheric concentration, seasonality, distribution, and allergenicity [55]. Thus, practical measures to limit global warming by reducing and curbing greenhouse gas emissions, which has implications in global temperature, is likely to have a positive health impact in reducing exposure to air pollution as a whole, and aeroallergens in particular, both in indoor and outdoor settings.

In terms of pollen speciation in indoor environments, about 50% of the studies included in this brief review provide data on the different types of pollen (e.g., *Betula, Poaceae, Urticaceae, Ambrosia, Carpinus Platanus, Quercus, *and* Cupressaceae* pollen). Besides data on pollen count, it is advisable to include the identification of pollen grains in studies on indoor exposure to pollen, not only to acquire more complete knowledge of the overall indoor air quality, but also to assess the impact on pollen allergy sufferers [21]. As a whole, the composition of pollen indoors varies throughout the year, especially in dust [45], and is also influenced by season [45, 46, 49]. Furthermore, global climate change scenarios (namely, the increase in CO_2_ levels and air temperature, as well as drought stress) may also affect the growth of plants and, consequently, the prevalence and distribution of the different pollen taxa and their allergenicity [56, 57]. According to Pichot et al., the pollen type, alongside housing and sampling time period, have an impact on the penetration and remanence of pollen grains indoors [52]. Moreover, pollen allergy sufferers seem to be exposed indoors to a similar and richer pollen spectrum compared to that outdoors, but in a much lower quantity [45]. Thus, in terms of allergen avoidance in indoor settings, it is crucial to collect information on the different pollen entities, which is even more important for polysensitized humans, who exhibit diverse reactions and, subsequently, symptoms to different types of pollen [21]. It should be mentioned that the analysis of pollen grains is still a challenging task, often relying on the use of a microscope and a trained specialist. To tackle this challenge and improve taxonomic resolution of pollen in aerobiological samples, neural networks mathematical tools have been successfully used to distinguish morphologically similar pollen from the same plant family [58]. The study by Polling et al. [58] demonstrated the advantage of using chemometric tools (in this case, sufficiently trained deep learning models) to distinguish pollen genera that exhibit different allergenic profiles, but which are indiscernible with the available microscopic methods. Acquisition of such data in a more automated fashion has a huge significance in terms of human health, since current measures to minimize exposure to allergenic pollen indoors can be improved.

### Quantification of allergenic pollen

Indoor pollen concentrations strongly depend on the ventilation type (forced or natural ventilation), building location, presence of indoor ornamental plants, window opening behavior, as well as window orientation towards the dominant wind direction [27–29, 50]. Urban rooftop greenhouses can also contribute to high levels of pollen grains and fungal spores in built environments [46]. This can pose serious problems if using bidirectional integrated rooftop greenhouse systems [38] that allow air to be drawn from the crop area into the building spaces. In such cases, improving air quality in the greenhouse for building users requires not only close monitoring of the biological air quality, but also use of preventive measures whenever appropriate (e.g., monitoring of the ventilation system and appropriate air filter systems) [46]. It has been also shown in dedicated studies that indoor environments without air purification devices are characterized by higher indoor pollen loads [36, 38, 43, 45]. Portable room air purification devices equipped with high efficiency particulate air (HEPA) filters exhibit a particulate matter reduction efficiency—depending on the particle size fraction—on the order of 50–70% [59–61]. In addition to building-specific and equipment-specific aspects, the occupant’s activity (either indoor or outdoor) also affects indoor pollen loads as building occupants may act as vehicles in the spread of pollen and fungal spores. For example, pollen or fungal spores can adhere to the occupants’ clothes or already settled pollen and fungal spores are remobilized by the occupants [33, 34, 38, 42, 50]. Pollen grains, in turn, can act as carriers for airborne virus particles or for airborne infected saliva droplets. Thereby, airborne virus particles adhering to pollen grains may increase virus transmission rates in indoor environments [35].

Typically, the indoor/outdoor concentration ratio is reported to assess the effect of infiltration of outdoor airborne trace constituents into indoor environments. With respect to airborne pollen, it could be also shown that the indoor/outdoor concentration ratio is affected by meteorological conditions, such as rainy episodes, temperature/season, and wind speed and direction [28, 39, 51–54]. For example, with higher temperatures, indoor pollen concentrations and indoor/outdoor ratios increase, not only mirroring higher pollen shedding, but also suggesting efficient transport during sunny days [50]. In a similar fashion, indoor pollen content tends to be lower in winter than in warm/dry seasons, correlating with both the flowering season and the individual cleaning/ventilation habits (i.e., opening of doors and windows in warmer seasons facilitates the penetration of pollen into dwellings) [52–54]. On the other hand, during rainy episodes, wash-out of airborne pollen outdoors and accumulation of airborne pollen indoors results in increased indoor/outdoor concentration ratios [28, 39]. Although increased indoor/outdoor pollen concentration ratios are not necessarily linked to increased allergy symptom loads indoors, special attention should also be given to fungal spores. In a study conducted in various occupational settings (market, saw mill, agriculture) [54], the presence of Amerospores, *Cladosporium, Ganoderma*, and *Nigrospora* were reported as the dominant fungal spore components, some of which are highly allergenic. According to Eduard [62], respiratory symptoms and airway inflammation in nonsensitized working populations are likely to appear at exposure levels as low as 105 spores/m^3^ for diverse fungal species. Although this is not an established threshold concentration for indoor environments, it seems that fungal spores may elicit allergic symptoms even in small quantities. As such, it can be concluded that besides reporting indoor/outdoor concentration ratios for pollen grain and fungal spores, monitoring surveys should also include absolute concentration values to better assess the prevailing pollen and fungal spores loads and the related health risks.

### Correlation with allergy symptoms

Undoubtedly, associating exposure to indoor pollen grains and/or fungal spores and allergy symptoms and health problems is challenging, but also the main reason for designing and implementing adequate indoor pollen monitoring strategies. The diverse factors affecting indoor pollen/fungal content (i.e., building type, indoor ambient conditions, characteristics of the outdoor surroundings, and meteorological/seasonal conditions), the variability associated with the pollen and fungal spore types and corresponding exposure studies and, consequently, finding the most adequate experimental design and standard analytical procedure (including sampling), make indoor pollen/fungal exposure studies very difficult. Furthermore, pollen/fungal allergies are either underdiagnosed or maltreated, which hinders the implementation of specific measures to manage and cope with airborne indoor allergens. Therefore, it is with no surprise that health aspects associated with exposure to these indoor allergens are still poorly understood. With regard to allergy symptoms, studies have reported an association between inhalant allergens (e.g., timothy and birch) and asthma and allergic rhinitis in children (aged 6–18 years) [44], and also the development of nasal, ocular, and bronchia irritation upon exposure to grass pollen or *Artemisia* pollen [41, 43, 47]. The clinical importance of allergens has been also determined by the frequency and intensity of their IgE antibody binding (allergenicity). In addition, some pollen allergens also induce inflammation through non-IgE-mediated pathways, which can increase their allergenic activity. However, for most indoor allergens (including pollen allergens), this important aspect has not been investigated and, therefore, deserves to be included in indoor allergens exposure studies [37].

It is also worth mentioning that family rhinitis history and plant pollen triggering are among the top contributing factors for asthma and allergy-related symptoms [30]. As such, individual allergy history is a major risk factor that should be taken into account when assessing sick building syndrome [31]. Besides the genetic factor, the environment in which a person is living might also be an important feature for the development of allergies. A questionnaire-based study on the self-reported pollen allergy prevalence indicates that individuals with occupational exposure to pollen may be at lower risk than indoor workers [26]. Within this scenario, where indoor workers, particularly those in urban areas, might be especially affected by pollen allergies, it becomes fundamental to measure and understand health stress response mechanisms to pollen exposure in indoor air.

## Conclusions and perspectives

Indoor environments received more attention with regard to airborne allergenic pollen. The studies published in the last 10 years have shown that controlling allergies begins in the built environment—in both private residences and occupational settings. Most people exhibiting allergy symptoms spend most of their time indoors, particularly when the atmospheric levels of pollen grains are high in the outdoor surroundings. Therefore, besides the initial driving question of this work (i.e., “What is the relevance of airborne allergenic pollen in indoor environments?”), one should also consider “How to facilitate monitoring of airborne allergenic pollen indoors?”

The outcomes of this brief review recommend further investigations with special focus on:

- The measurement location: research about trends in airborne pollen indoors has focused on limited building settings, whereby the results varied regarding pollen loads and pollen types. Therefore, systematic analyses of allergenic pollen indoors across different geographical areas and in different built environments are urgently needed.
- The measurement period: Within the locations/sites studied thus far, the results have shown an increase in indoor pollen levels with flowering season, whereby the meteorological conditions also play an important role in the observed pollen loads. Moreover, a change in seasonal duration due to the current and projected climate change scenarios (i.e., temperature increases, higher CO_2_ levels, and drought stress) is likely to have a positive correlation with pollen load indoors and, therefore, an impact on the allergenic potential of pollen. Hence, systematic long-term analyses of allergenic pollen indoors, merging different locations, built environments and seasons are required to better assess indoor exposure on a global scale.
- Speciation of allergenic pollen: Understanding the most important allergenic pollen taxa in built environments requires large-scale coordinated studies assisted by chemometric tools, taking into account not only the regional nature of plant behavior in the outdoor surroundings, but also the local climate. Such an approach would allow the characterization and mapping of the spatial and temporal variability of the allergenic pollen taxa indoors, thus, facilitating the design and adoption of measures to tackle the health impacts related to pollen allergies in confined spaces.
- The quantification of allergenic pollen: Assessing the aeroallergen pollen concentrations indoors is an important metric of medical significance. Understanding allergenic pollen concentrations, alongside their identity, in built environments is of utmost importance to better ascertain and project health consequences. Ultimately, the acquired knowledge will be crucial to develop a coordinated indoor air quality strategy, with a focus in monitoring and reducing biologic pollution in confined spaces.
- The investigation of symptom loads: Gathering complementary data on the prevalence of allergies for different groups of building occupants along with the speciation and quantification of allergenic pollen will help to build a knowledge base for assessing the relevance of associated health effects. Furthermore, this approach will help to identify the onset of allergy symptoms and health effects and thereby provide tools for the enforcement of improved technical standards as well as regulatory measures. Finally, the systematic investigation of symptom loads along with concentration loads of airborne allergenic pollen in the built environment will provide information for the assessment of the corresponding global burden and thus of the cost for national healthcare systems.

To the best of the authors’ knowledge, a coordinated strategy addressing the above-mentioned specific objectives has not been done to date, in part because there are no globally accepted standard operation procedures for data collection and data processing, and there is no major global repository for the acquired information. Nonetheless, such data will allow a broader basis to be established to make correlations between pollen loads and symptom loads in built environments. This will further enable epidemiological assessments to facilitate the identification of the most susceptible building occupants suffering from allergenic symptoms. Complementary citizen–science approaches might also support the generation of a common database. Such an involvement of the general public might comprise active sampling (e.g., visual inspection of samples from air cleaning devices or vacuum cleaners) as well as passive sampling (e.g., visual inspection of samples from passive traps or pollen screens) to assess the occurrence of pollen in residential environments. The use of pollen forecasts (e.g., using dedicated apps) might support such citizen–science approaches.

Finally, all the measures outlined above will assist the design of promising mitigation measures to prevent large pollen loads and, consequently, symptom loads indoors.
